# A Temporary Acrylic Soft Denture Lining Material Enriched with Silver-Releasing Filler-Cytotoxicity, Mechanical and Antifungal Properties

**DOI:** 10.3390/ma17040902

**Published:** 2024-02-15

**Authors:** Grzegorz Chladek, Igor Kalamarz, Wojciech Pakieła, Izabela Barszczewska-Rybarek, Zenon Czuba, Anna Mertas

**Affiliations:** 1 Materials Research Laboratory, Faculty of Mechanical Engineering, Silesian University of Technology, 18a Konarskiego Str., 41-100 Gliwice, Poland; 2 Igor Kalamarz Dental Practice, 6 Kotlarza Str., 40-139 Katowice, Poland; i.kalamarz@gmail.com; 3 Department of Engineering Materials and Biomaterials, Faculty of Mechanical Engineering, Silesian University of Technology, 18a Konarskiego Str., 41-100 Gliwice, Poland; wojciech.pakiela@polsl.pl; 4 Department of Physical Chemistry and Technology of Polymers, Faculty of Chemistry, Silesian University of Technology, Strzody 9 Str., 44-100 Gliwice, Poland; izabela.barszczewska-rybarek@polsl.pl; 5 Department of Microbiology and Immunology, Faculty of Medical Sciences in Zabrze, Medical University of Silesia in Katowice, 19 Jordana Str., 41-808 Zabrze, Poland; zczuba@sum.edu.pl (Z.C.); amertas@sum.edu.pl (A.M.)

**Keywords:** temporary denture soft linings, dental materials, silver, antifungal properties, cytotoxicity, functional properties

## Abstract

Colonization of temporary denture soft linings and underlying tissues by yeast-like fungi is an important clinical problem due to the negative influence on the process of prosthetic treatment. Typical hygienic procedures are often insufficient to prevent fungal infections, so in this study, an antimicrobial filler (silver sodium hydrogen zirconium phosphate) was introduced into acrylic soft liner at concentrations of 1, 2, 4, 6, 8 and 10% (*w*/*w*). The effect of this modification on antifungal properties against *Candida albicans*, cytotoxicity, Shore A hardness, tensile strength and tensile bond strength, sorption and solubility was investigated, considering the recommended 30-day period of temporary soft lining use. The most favorable compilation of properties was obtained at a 1 to 6% filler content, for which nearly a total reduction in *Candida albicans* was registered even after 30 days of sample storing. The tensile and bond strength of these composites was at the desired and stable level and did not differ from the results for the control material. Hardness increased with the increasing concentration in filler but were within the range typical for soft lining materials and their changes during the experiment were similar to the control material. The materials were not cytotoxic and sorption and solubility levels were stable.

## 1. Introduction

Soft and elastic materials bonded to the surface of dentures or some other prosthetic devices in the oral cavity in order to reduce their traumatic impact on the underlying tissues are defined as denture soft linings. Their application allows for a more uniform distribution of forces transferred to the tissues under prosthesis and dissipation of some energy during work [1]. This plays a protective role mainly in relation to soft tissues because it helps relieve the mucosa from high local loads, which supports the prosthetic treatment process [2]. In ISO 10139 standards, soft relining materials are classified as long-term (more than 30 days) or short-term (a continuous period from 60 min up to 30 days), where the second group is clinically used as tissue conditioners and as temporary soft lining materials [3,4]. Tissue conditioners are plasticized, uncross-linked, viscoelastic gels characterized by a rapid loss of physicochemical properties [5,6] and placed on the surface of the prosthesis for a period of up to 7 days to restore the mucosa to a proper, healthy state [4]. Temporary soft lining materials are usually a cross-linked acrylic that are supplied by manufacturers as two-component “powder-liquid” systems, where “powder” is prepolymerized poly(ethyl methacrylate) (PEMA) with the initiator and ”liquid” consisted mixture of plasticizers (low molecular weight esters, such as dibutyl phthalate or ethyl acetate) with monomers such as methyl ethyl or butyl methacrylates, characterized by a longer side chain length, which reduces the glass transition temperature and hardness [7]. The properties of cross-linked materials are much more stable than for tissue conditioners [2]; however, they are still worse than for long term soft linings, especially when we take into account the tendency for hardening [8] and the values of sorption and solubility, which are too high to classify them as long-term materials [9]. It should be noted that ISO standards do not indicate any differences in physicochemical properties for tissue conditioners and other short-term soft liners, and moreover, cross-linked temporary soft liners are often used in place of tissue conditioners in cases where it is not practicable to replace the conditioner every few days [6]. Due to the low modulus of elasticity and the ability to dissipate energy during chewing, temporary relining materials are used in clinical practice for relining immediate dentures, in case of mucosa pain associated with the impact of a hard prosthesis, during implantological treatment and during treatment of mucosal injuries resulting, for example, from surgical procedures or denture function, to temporarily improve the fit of an ill-fitting denture until the development of a new one or as a diagnostic aid to determine whether the patient would prefer to use a long-term soft lining [10,11]. Classic soft lining materials belong to the group of polymeric dental materials, but clinical challenges make it necessary to take a creative approach and search for new solutions that will improve their properties. This progress can be achieved, as in the case of other types of dental materials, by developing composites containing a reinforcing phase that enhances the desired biofunctional features [12] and by a conscious design based on modern methodology [13].

Maintaining proper hygiene of dentures is essential for the success of prosthetic treatment, especially in the case of wounds of the mucosa; however, it is difficult if conditions in the oral cavity (temperature, humidity, low pH value, closed space between the mucosa and the prosthesis favoring the growth of pathogenic microflora) are considered [14]. Colonization of soft lining materials, mainly by yeast-like fungi such as *Candida albicans* (*C. albicans*), increases the risk of complications during treatment and limits its effectiveness. These microorganisms have been shown to colonize the surface of materials, but also to penetrate inside them [15]. Research indicates that hydrophilic acrylic soft linings show increased susceptibility to colonization [16], and the problem cannot be solved by the use of denture cleaning agents, which cause surface microdamage that increases the adherence of *C. albicans* and favors the colonization process [17]. For this reason, investigations focused on increasing the antimicrobial resistance of soft linings are considered particularly important. In the case of tissue conditioners, the solution most frequently proposed is the combination of treatment with pharmacotherapy [18] or the use of additives in the form of antibiotics [19] or natural oils [20,21] to materials, but these solutions are not effective in the case of prolonged use of other than tissue-conditioner short-term soft linings due to the leaching of active substances and increasing drug resistance of microorganisms [22]. It is believed that obtaining a more sustained antimicrobial effect without the risk of developing drug resistance is possible by introducing fillers with antimicrobial properties or by adding polymeric ingredients. It has also been shown that an antimicrobial effect can be achieved as a result of the use of magnesium oxide [23] or chlorhexidine nanoparticles [24], and chemical modification of the surface by long chain (hydrophilic or hydrophobic) can reduce the initial adherence of *C. albicans* [25]. However, in those investigations, only the initial antifungal effect (without stability in time) was tested, and the influence of these additives on other materials’ properties was not investigated. Special attention was paid to the addition of nanosilver. It was tested as an additive to long-term soft lining [26] or tissue conditioners [27]. For long-term materials, the stability of the antifungal effect was not tested [26], but for tissue conditioner, the antimicrobial effectiveness was obtained for 72 h, but only at high concentrations [27]. However, the introduction of nanosilver into soft lining was the cause of a decrease in mechanical properties [26]; moreover, a significant color change (dark discoloration) due to the plasmon effect on nanoparticles was observed [28] which is unacceptable for to practical reasons. Ferreira et al. [29] investigated the antifungal efficacy of silver-zinc zeolite nanoparticles incorporated into a temporary soft lining during the 30-day experiment and achieved some success whereby after a week the antimicrobial effect gradually decreased over time. The study did not analyze any properties other than microbiological. Kreve et al. [30] investigated the effect of nanostructured silver vanadate decorated with silver nanoparticles and showed an initial antifungal effect and an ambiguous effect (increase or decrease depending on the resin used) on tensile bond strength to denture base resins, but the stability of investigated properties was not tested.

The studies conducted so far indicate that the use of silver may contribute to the solution of microbiological problems related to the use of temporary soft linings; however, it is necessary to search for additives that will eliminate the unfavorable consequences of introducing, e.g., nanosilver. Such a carrier may be silver-sodium-hydrogen-zirconium phosphate, which has not been studied in this regard to date. The use of this white-in-color ceramic carrier, which releases silver ions to the environment, seems to be of particular interest, but it is necessary to determine the microbiological effect and mechanical properties in the expected life of the materials in the first stage of investigations. It should be emphasized that previous research on the use of silver or silver carriers focused on the assessment of microbiological properties in the initial state (with one exception), and the mechanical properties were usually not analyzed or analyzed only in the initial state. The aim of the present study was to investigate the antifungal and mechanical properties and their stability during the expected period of the use of experimental composite material for the temporary relining of dentures filled with silver-sodium-hydrogen-zirconium phosphate. The thesis was that it is possible to obtain increased antimicrobial resistance of the acrylic soft lining material by using a submicrometer filler in the form of silver-sodium-hydrogen-zirconium phosphate, while maintaining the advantageous properties of the starting material. 

## 2. Materials and Methods

### 2.1. Materials Preparation

Vertex Soft (VS) (Vertex Dental, The Netherlands), a two-component (powder-liquid system) acrylic, declared by the manufacturer as temporary soft relining, was used as the modified material and the control material (CM) is the component heat-cured system where pre-polymerized powder is composed of poly(ethyl methacrylate) and benzoyl peroxide and liquid is a mixture of plasticizer (>80% *w*/*w*), methyl lmethacrylate (<20% *w*/*w*) and ethylenglycol dimethacrylate (<5% *w*/*w*). An antimicrobial filler (AF) was silver-sodium-hydrogen-zirconium phosphate with the total formula Ag_0.46_Na_0.29_H_0.25_Zr_2_(PO_4_)_3_ (Milliken Chemical, Spartanburg, SC, USA) containing approximately 10% silver (by weight) [31]. 

**Figure 1:**
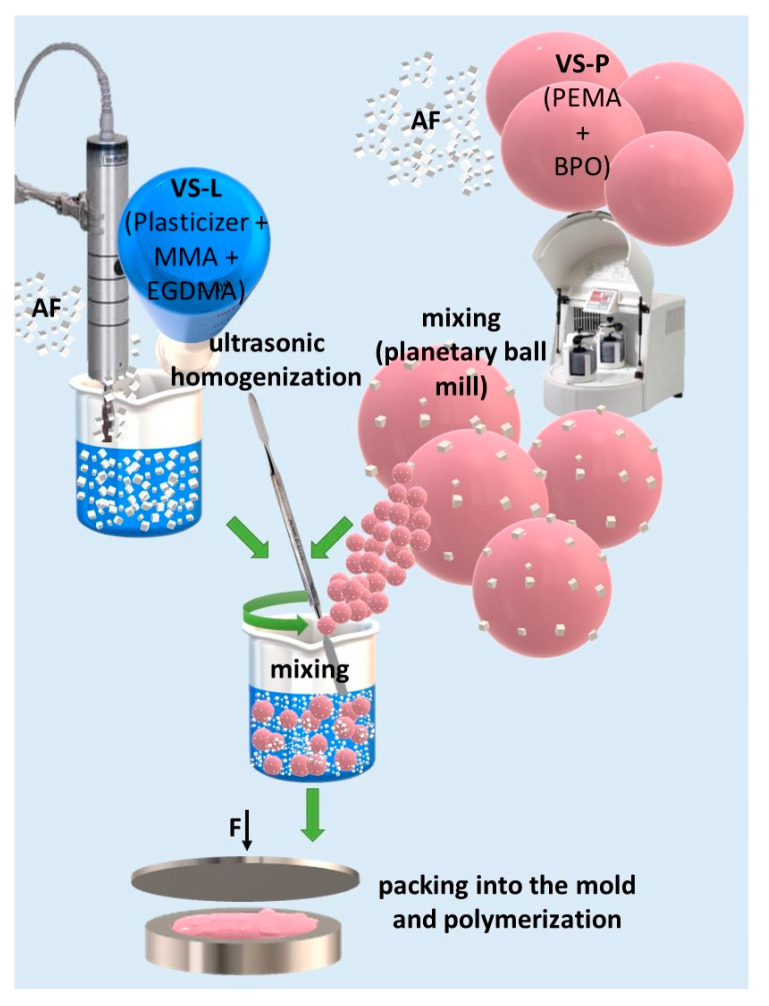
A schematic presentation of the composites preparation procedure; AF—antimicrobial filler, VS-L—Vertex Soft liquid component, VS-P—Vertex Soft powder component, MMA—methyl lmethacrylate, EGDMA—ethylenglycol dimethacrylate, PEMA—poly(ethyl methacrylate), BPO—benzoyl peroxide.

Figure 1 shows a schematic presentation of the composites’ preparation procedure. The filler was introduced into the material in two stages. The concentration of AF in the Vertex Soft powder (VSP) component was constant and limited to 1% because the use of the higher concentrations promotes the formation of an aggregation of filler particles on the surface of the particles of the modified powders [32]. AF to VS-P was mixed using a planetary ball mill (Pulverisette 5, Fritsch, Idar-Oberstein, Germany). The mixing time of 5 min with a rotation frequency of 400 rpm were used to introduce filler into 10 g of VS-P in a grinding bowl (volume 250 mL) with 50 pieces of 10 mm diameter ZrO_2_ balls; these parameters were determined in previous experiments [32]. All mass measurements were carried out on a Radwag AS/X analytical balance with an accuracy of 0.001 g. The material obtained in this form was suitable for long-term storage.

The mass concentration of AF in the Vertex Soft liquid (VSL) component was calculated to obtain the final mass concentration in composites of 1, 2, 4, 6, 8 and 10% after polymerization of the samples (Table 1) taking into account the proportions recommended by the manufacturer in which VSL and VSP components should be combined. Modified VSL was obtained with the use of a constant mass of the liquid (15 g) to keep the experimental conditions unchanged (identical conditions of combining VSL with AF). The weighed mass of VSL was placed in a glass test tube, then AF was introduced into it, the test tube was closed and shaken for about 30 s. The test tube was placed on a rack, immersing it in water to cool the system (temperature 16 to 18 °C) and subjected to ultrasonic homogenization in three sequences for a total of 90 s (Ultrasonic Homogenizer UP200St, Hielscher Ultrasonics, Teltow, Germany).

The samples were polymerized according to the manufacturer’s instructions immediately after ultrasonic homogenization was completed to prevent sedimentation of the AF in VSL. During the mixing of the components, the manufacturer’s recommended proportion of unmodified VSP to VSL was followed (VS-L/VS-P ratio is 1.273). The necessary mass of the materials was calculated on the volume of the molds in which polymerization was carried out, assuming at least 50% material loss during manual activities. The mass of the modified materials for making the mixtures was calculated from the equations:

(1) $$m_{VSPAF}=1.010101\times m_{VSP}$$

(2) $$m_{VSLAF}=m_{VSL\;}-\left(\frac{C_{AFVSL\;}\times m_{VSL\;}}{1-C_{AFVSL\;}}\right)$$

where: *m_VSPAF_*—mass of VSP with the addition of 1% AF, g; *m_VSP_*—mass of *VSP* necessary to obtain the material with the proportions of *VSL* to *VSP* recommended by the manufacturer, g; *m_VSLAF_*—mass of *VSL* with the addition of AF, g; *m_VSL_*—mass of *VSL* necessary to obtain the material with the proportions of *VSL* to *VSP* recommended by the manufacturer, g; *c_AFVSL_*—concentration of *AF* in the *VSL* component, %.

Test samples (excluding bond strength tests) were polymerized in stainless steel (316 L) molds using an insulator (samples for hardness tests) and/or spacers made of Teflon foil (other samples). The internal diameter of the mold for samples for hardness testing was 40 mm and its height was 6 mm. For scanning electron microscopy (SEM), microbiological tests and tensile strength tests 80 × 80 mm and 2 mm thick plates were made from which samples of appropriate shapes were cut.

### 2.2. Methods

#### 2.2.1. Scanning Electron Microscope Investigations

The qualitative assessment of VSP morphology after mixing with AF and the qualitative analysis of AF particle dispersion in the composites were carried out with scanning electron microscopy investigations (SEM) (Supra 25, Zeiss, Berlin, Germany). For the powder samples, they were taken from randomly selected locations and placed on a carbon tape (Agar Scientific, London, UK). Filler dispersion in the polymerized composites was analyzed in cross sections obtained by breaking the sample previously cut to 1/3 of its thickness in liquid nitrogen. The samples were dried for 2 h at a temperature of 40 ± 1 °C in a desiccator containing silica gel freshly dried at 130 °C for 4 h. The samples were sputtered with gold. The accelerating voltage was 1 to 5 kV.

#### 2.2.2. Antifungal Efficacy

Rectangular samples that measured 10 × 10 × 2 mm were stored in 500 ± 20 mL of distilled water (Avantor, Gliwice, Poland) for 24 h, 7 days and 30 days at 37 ± 1 °C. The water was changed every 3 days. After storage, the samples were dried at 37 ± 1 °C for 48 h in desiccators containing dried silica gel. The antifungal properties tests were carried out based on the previously described method [33]. Standard strains of *Candida albicans* ATCC 10231 (*C. albicans*) were used. Sterilized square samples were immersed individually in 1 mL of *C. albicans* suspensions containing approximately 1.5 × 10^5^ CFU/mL (CFU, colony-forming units) in tryptone water. *C. albicans* was tested as a positive control, pure tryptone water was tested as a negative control. The samples were incubated in a shaking incubator for 17 h at 35 °C for *C. albicans* and then 20 μL of suspension was seeded in Sabouraud agar plates (bioMerieux, (Marcy l’Etoille, Lyon, France). Cultured plates were incubated at 35 °C for 17 h, the colonies were counted and composite antifungal efficacy of the compounds was calculated:

(3) $$AFE=\frac{V_{c}-V_{t}}{V_{c}}\times 100\%$$

where V_c_ is the number of viable colonies of the positive control (BLANK), CFU/mL, V_t_ is the number of viable colonies of the test specimen, CFU/mL.

#### 2.2.3. Adherence of *Candida albicans* Cells

Fungal adhesion tests were performed by incubating rectangular samples that measured 10 × 10 × 2 mm stored in 500 ± 20 mL of distilled water (Avantor, Gliwice, Poland) for 24 h and 30 days at 37 ± 1 °C. The water was changed every 3 days. After storage, all samples were placed for 18 h in 1 mL of *C. albicans* ATCC 10231 suspension ~1.5 × 10^5^ CFU/mL in tryptone water at 37 °C and the methodology described in the work [34] was then used with modifications regarding the substrate used to determine cell adherence. The samples were vortexed in 1 mL of sterile water, 100 µL of undiluted obtained suspensions were seeded onto Sabouraud agar plates (bioMerieux) (Marcy l’Etoille, Lyon, France) and incubated at 37 °C for 24 h. The number of cells was measured by counting the colonies (automatic colony counter ProtoCOL 3 PLUS, Synbiosis, Frederick, MD, USA).

#### 2.2.4. Cell Viability Assay (MTT Assay)

The cell viability assay was performed in accordance with the EN ISO 10993-5:2009 standard [35] and the methodological details were fully described in previous work [34]. Rectangular samples that measured 10 × 10 × 2 mm of the composites were placed in 2 mL of culture medium used for the culture of fibroblasts of the L-929 line and incubated at 37 °C in an atmosphere of 5% CO_2_ for 2 or 10 days (2-day and 10-day extracts). Culture medium incubated at the same conditions was a control. A suspension of cell culture of the L-929 line (NCTC clone 929) purchased from the American Type Culture Collection, catalogue number CCL-1 (Manassas, VA, USA) with a final density of 1 × 10^5^ cells/mL of medium was used. Finally, the viabilities of L-929 cells contacted with the extracts of the tested composites were evaluated. Mouse fibroblasts under in vitro culture conditions were incubated for 24 h with undiluted extracts and their viability was assessed using the bromo-3-[4,5-dimethylthiazol-2-yl]-2,5-diphenyltetrazolium assay (MTT assay).

The DMSO was used to extract MTT formazan. The absorbance was determined at 550 nm using the Eon automatic plate reader (BioTek Instruments, Winooski, VT, USA). Cell viability (%) was calculated using the formula:

(4) $$Cell\;viability=\frac{Ab}{Ak}\times 100\%$$

where: *Ab*—the absorbance of the test sample, *Ak—*the absorbance of the control.

#### 2.2.5. Shore A Hardness

For Shore A hardness measurements 3 samples (40 mm in diameter and 6 mm in thickness) for each material were prepared and values were registered after 5 s of loading at 5 points of every sample as recommended in ISO 48-4:2018 [36] standard with digital durometer (Bareiss HPE II-A, Bariess, Oberdischingen, Germany). Tests were performed after 24 h, 7 days and 28 days of storing of samples in distilled water at 37 ± 1 °C. To take into account the influence of temperature on the hardness values of this type of material, 2 h before measurements, the samples were placed in steel molds that facilitated the maintenance of the temperature after being removed from the bath. After removing the sample from water, it was rapidly dried of visible moisture with filter paper, 3 measurements made within a maximum of 30 s, and the sample was placed in water for another 10 min, followed by the remaining 2 measurements.

#### 2.2.6. Tensile Strength 

The dumbbell-shaped samples of type 4 specified by ISO standard [37] were stored in distilled water at 37 ± 1 °C (24 h, 7 days and 28 days). For each composite/conditioning time, 10 samples were made. After storing, the samples were measured and tensiled with a cross-head speed of 10 mm/min until brake using a universal testing machine (Zwick Z020, Zwick GmbH & Com, Ulm, Germany). The ultimate tensile strength was calculated according to the equation:

(5) $$T_{s}=\frac{F}{A}$$

where: T_S_—ultimate tensile strength, MPa; F—force at rupture, N; A—initial cross-sectional area, mm^2^.

Randomly selected fractures were sampled with gold and investigated with SEM at the accelerating voltage of 1 to 5 kV.

#### 2.2.7. Tensile Bond Strength

Tensile bond strength (T_B_) to the denture base resin was examined according to the method presented in [3,33] with additional and necessary specifications for the preparation of samples. Samples of PMMA denture base resin (DBR) (Vertex RS, Vertex-Dental B.V., Zeist, The Netherlands) measuring 20 × 20 mm and 3.3 ± 0.2 mm thick were cured in accordance with the manufacturer’s instructions, wet ground with P500-grit abrasive paper to standardize the surface, air blasted with Al_2_O_3_ (110 µm) particles at 4 bar pressure and finally cleaned in an ultrasonic cleaner for 10 min. Dentures of the base material samples were conditioned in distilled water (37 ± 1 °C, 28 days), and then removed from water in pairs and dried. The control and experimental soft lining materials were placed in a polyethylene ring (inner diameter 11 mm, height 3 ± 0.1 mm) and compressed between two samples of denture base material, inserted between two brass flat bars that were twisted to apply pressure as is the case with the flasking method in a standard prosthetic procedure. After polymerization was conducted according to the manufacturer’s instructions, the samples were placed in distilled water at a temperature of 37 ± 1 °C for 24 h, 7 days, and 30 days. Ten samples were prepared from each composite. Two to three hours before the end of the storage time, the samples were removed from the water with handles in the form of M4 using reducers as it was described in the paper [33]. The samples were placed back in the water until the anticipated conditioning time was complete and finally were removed from the water, mounted in reducers in the jaws of the testing machine and a tensile test was performed at a cross-head speed of 10 mm/min. Tensile bond strength was calculated with the equation:

(6) $$T_{B}=\frac{F_{m}}{A}$$

where: T_B_—tensile bond strength, MPa; F_m_—maximal force, N; and A—the initial cross-sectional area (internal area of the polyethylene ring), mm^2^.

For each of the samples, the type of fracture was determined and classified as follows.

- Type A: debonding of the material from the denture base material (adhesive fracture) with the possible presence of remnants of the soft lining material invisible to the naked eye and not protruding from the plate.
- Type B—cohesive fracture: when only the soft lining was damaged (no zones indicating loss of bonding between the PMMA plate and the relining).
- Type A*—similar to type A: with the difference that there were single, visible with the naked eye, areas of cohesive destruction, where none of the dimensions exceeded 1 mm.
- Types A + B mixed fracture: when the fracture areas of types A and B were simultaneously represented.

Randomly selected fractures and interface morphologies between bonded materials obtained by breaking samples in liquid nitrogen were sputtered with gold and investigated with SEM at the accelerating voltage of 1 to 5 kV.

#### 2.2.8. Sorption and Solubility

Five samples (50 mm in diameter, 1 mm in height) of each material prepared in stainless steel molds were dried inside desiccators with freshly dried silica gel at 40 ± 1 °C and weighed daily (Analytic Scale AS 60/220.X2.PLUS, Radwag, Poland) with an accuracy of 0.1 mg to achieve daily changes in the mass < 0.1 mg (m_1_). Directly after drying the samples were placed individually in 100 mL of distilled water for 7 days at 37 ± 1 °C. After storing, the samples were dried from visible moisture with filter paper, weighed (m_2_) and the drying was repeated as previously (m_3_). The water sorption (WSO) and the solubility (WSL) were calculated according to the following formulas:

(7) $$\mathrm{WSO}=\frac{m_{2}-m_{3}}{m_{1}}\times 100\%$$

(8) $$\mathrm{WSL}=\frac{m_{1}-m_{3}}{m_{1}}$$

#### 2.2.9. Statistical Analysis

Statistical analysis of the results was performed using the PQStat version 1.6.6.204 (PQStat Software, Poland). The residuals distributions were tested with the Shapiro–Wilk test and the equality of variances was tested with the Levene test. One-way or two-way ANOVA with a possible F * correction (Brown–Forsythe) and Tukey HSD post hoc tests were used (α = 0.05) and the *t*-student test. The impact materials used in the fracture observed in the bond strength test were tested with the exact Fisher-Freeman-Halton test for tables R × C (α = 0.05). Antifungal efficacy test results were analyzed using non-parametric Kruskal–Wallis test (α = 0.05).

## 3. Results

**Figure 2:**
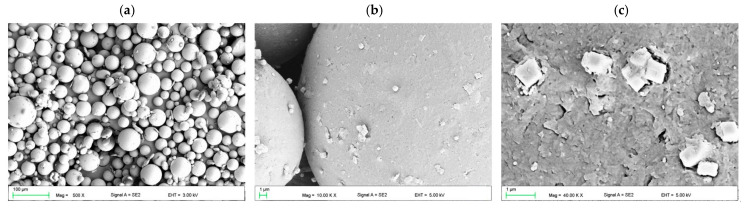
The prepolymerized spherical particles of VSP (**a**) and their surface after introducing 1% AF using a planetary ball mill (**b**,**c**).

The VSP particles were mostly 10 to 50 µm in diameter (Figure 2a). In Figure 2b,c, the surface of VSP with deposited AF particles was presented. Their number in relation to the entire surface of the VSP spheres was relatively small, which was consistent with the assumption, while the presence of both single AF particles and their small aggregations was observed. The filler dispersion on the AF particles was satisfactory. SEM observations also showed that a significant number of filler particles were pounded into the surface of the VSP particles (Figure 2c).

**Figure 3:**
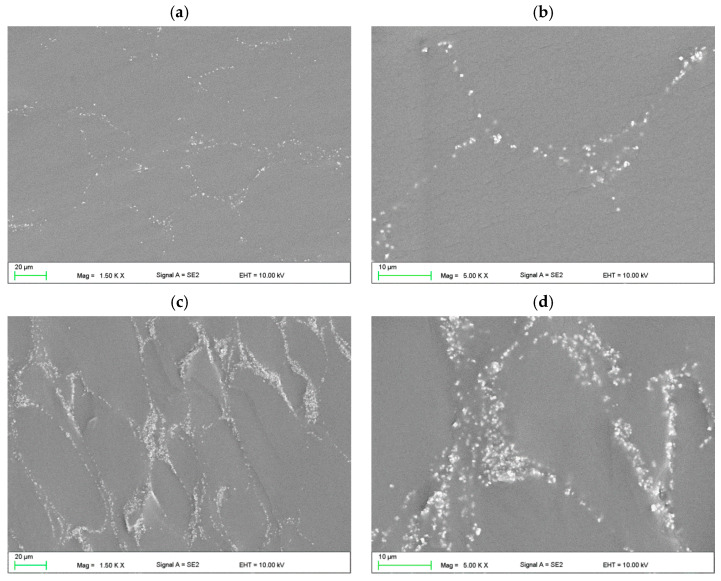
SEM microphotographs showing the morphologies of the cross-sectional area of polymerized C1 (**a**,**b**) and C6 (**c**,**d**) samples.

The morphologies after polymerization of the samples are presented in Figure 3. AF particles were distributed in areas between the prepolymerized spherical particles becoming of VSP. Single cubic particles and the aggregations of AF measuring 500 nm to 2 µm (composed of several dozen particles) were characteristic of composites from C1 to C4 (Figure 3a,b). Starting from C6, the distances between AF particles decreased and it is difficult to clearly state in what sizes the aggregations occur (Figure 3c,d). The areas between the particles were well filled with the polymerized material, no air bubbles or voids between the AF particles were observed. There was no tendency to increase the presence of the AF near the interface between VSP-derived particles and VSL-polymerized material. The SEM observations confirmed that the distribution of the filler may be determined as appropriate.

**Figure 4:**
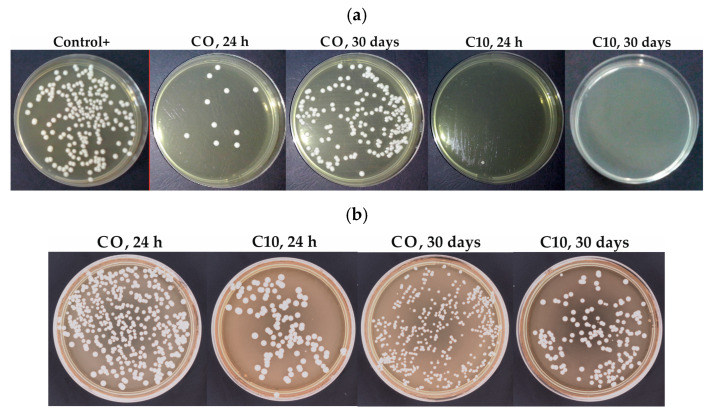
Representative images of cultured plates after incubation with suspensions of the *C. albicans* ATCC 10231 after antifungal tests (**a**) adherence tests (**b**).

The results of the antifungal test and their statistical analysis are presented in Table 2 and in Figure 4a. For samples stored in distilled water for 24 h and 7 days, statistically significant differences in Vt values (*p* < 0.05) were observed. The median value of Vt for CO was at least several times higher than that for composites. For all materials, high AFE values (reduction compared to positive control) were registered. For CO, the median was 95.1% after 24 h and 94.5% after 7 days, for composites, the median values ranged from 98.8 to 100%. After 30 days, the Vt values differed statistically significantly (*p* < 0.05). The median Vt value for CO was several dozen times higher than that for composites. For CO, the median AFE value was 25.5%, with the minimum value −24.4% (an increase in the number of microorganisms in relation to the positive control). Statistical analysis showed that in the case of CO, storage in distilled water caused statistically significant changes in Vt values, while for all composites, the differences were not statistically significant.

**Figure 5:**
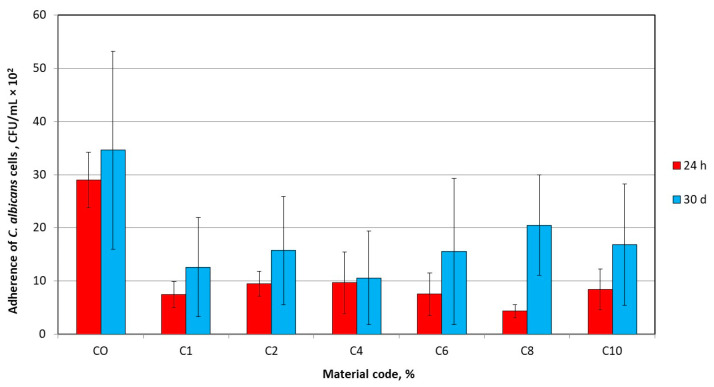
The number of *C. albicans* bacteria adhered to composites’ surface.

Initially, the number of live *C. albicans* adhered to the surfaces (Figure 4b and Figure 5) decreased significantly after the introduction of AF (*p* < 0.0001 and *p* = 0.0006 for samples stored in distilled water for 24 h and 30 days, respectively). For composites with AF, the number of adhered live cells was over three times smaller than for CO, but after 30 days, this difference was smaller with much higher standard deviation values. The post hoc tests did not show statistically significant differences in the number of adhered cells for composites with different AF concentrations. After 30 days, the number of live, attached *C. albicans* cells increased significantly (*p* = 0.0154) for CO. A similar tendency was noted for composites, but the differences were statistically significant only in the case of the two highest concentrations.

**Figure 6:**
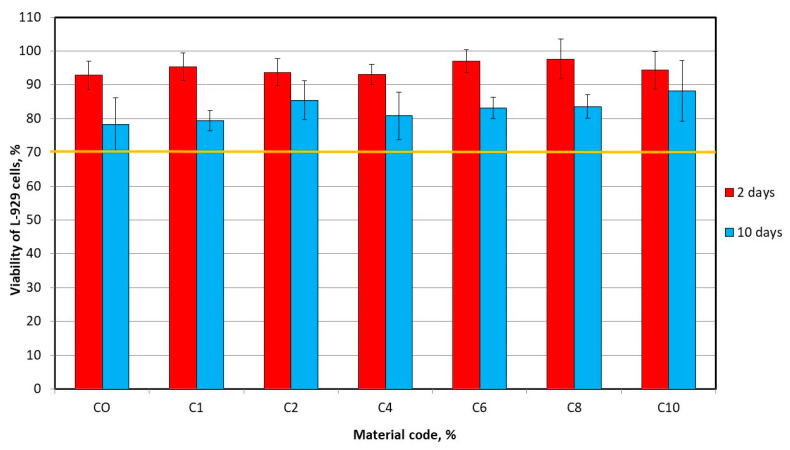
The viability of L-929 cells after 24 h of incubation with the 2-day and 10-day extracts.

The mean viability values of L-929 cells for the extracts are presented in Figure 6. For 2-day and 10-day extracts there was no statistically significant difference (*p* = 0.3895 and *p* = 0.0944, respectively) between the groups and all values were above 70%. The significant (*p* < 0.05) reduction in viability of cells due to the extension of extracting time were registered for materials most materials excluding C10, for which the reduction in viability was not statistically significant.

**Figure 7:**
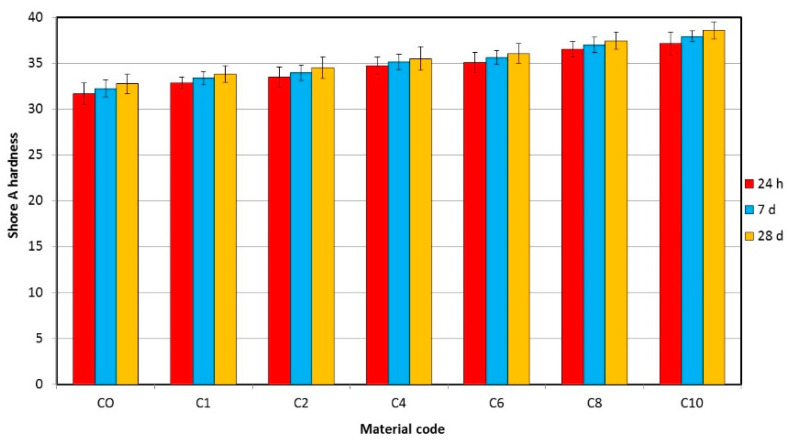
Mean hardness values with standard deviations for the tested materials.

The mean Shore A hardness values are presented in Figure 7. AF concentration and storage time significantly increase hardness values (Table 3); however, for two composite materials, the influence of storage time was not statistically significant. The mean hardness values after 24 h of storage in distilled water ranged from 31.7 (C0) to 37.1 (C10). After 30 days, the mean values for the C0 and C10 materials were 32.7 and 38.6 Shore A, respectively. The mean values for the C10 composite after 24 h and 30 days of storage were 17% and 18% higher, respectively, than for the material C0. The increase in hardness values with storage was from 2% (C4) to 4% (C10).

**Figure 8:**
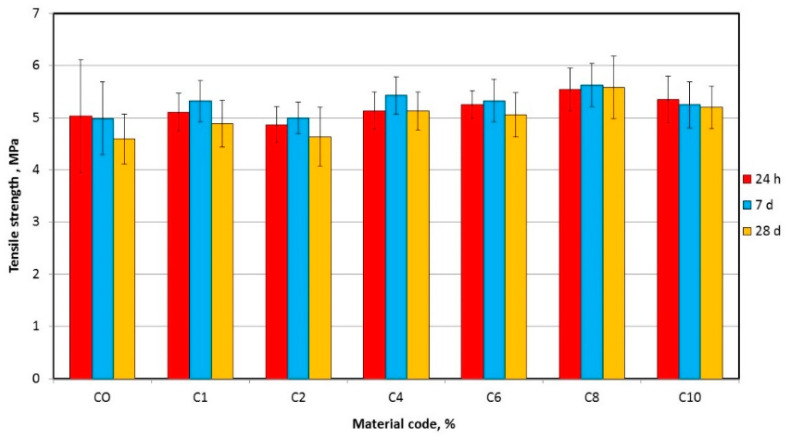
Mean tensile strength with standard deviations for tested materials.

The mean tensile strength values are presented in Figure 8. AF concentration significantly influenced mean tensile strength (Table 4); however, after 24 h of storage, differences were not statistically significant. After 7 days and 30 days of storage, the lowest mean tensile strength values were registered for C0 (5.0 MPa and 4.6 MPa, respectively), and the highest (5.6 MPa for both storage times) for C8. Storage time did not have a significant influence on tensile strength values.

**Figure 9:**
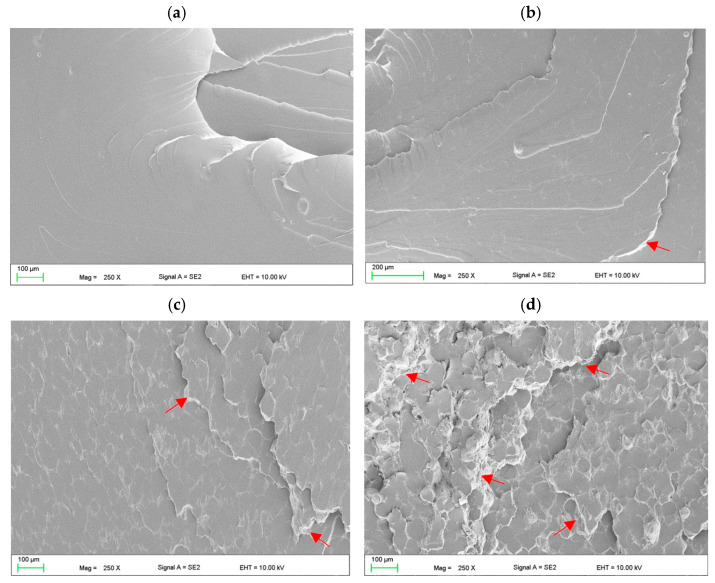
SEM microphotographs presenting the fracture morphologies after the tensile strength test for CO (**a**), C4 (**b**), C6 (**c**) and C10 (**d**).

SEM observations of fracture surfaces for materials from C0 (Figure 9a) to C4 (Figure 9b) showed similar morphologies; however, increasing the AF concentration increased the presence of areas of characteristic faults covered with AF (on samples indicated by red arrows) that indicate a local loss of load capacity of areas crated during polymerization of a modified VSL. Starting from the C6 (Figure 9c) to C10 material (Figure 9d), the shape of the faults became more irregular and their number and area increased.

**Figure 10:**
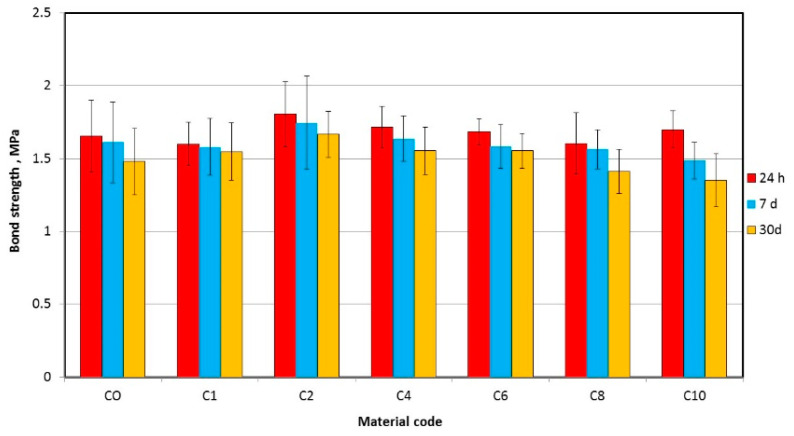
Mean tensile bond strength with standard deviations for tested materials.

**Figure 11:**
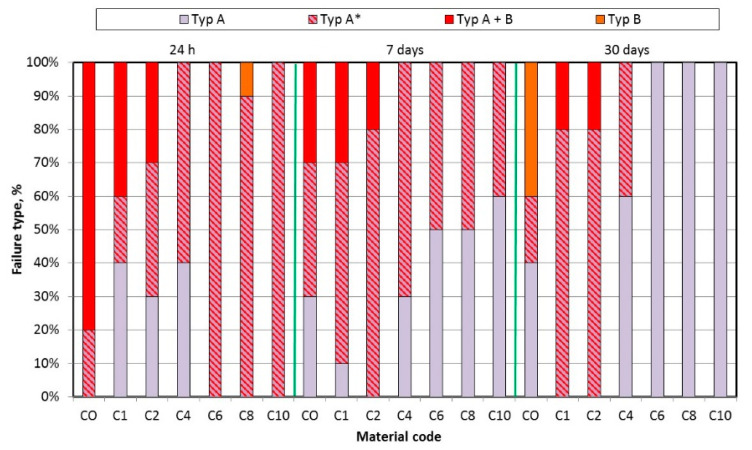
Impact of AF introduction on fracture type after bond strength tests.

The mean tensile bond strength values are presented in Figure 10. AF concentration significantly influenced mean TB values, but only after 30 days of storage (Table 5); storage time has a significant influence on the tensile bond strength values. For C8, statistically significant differences were recorded after 30 days compared to the starting point, but for the composite C10 the values differed, and statistically significant differences were observed after each storage time. The distributions of fracture types after bond strength tests are presented in Figure 11. The dominant type were A and A *, while the types A + B and B were observed, except for one sample, only for C0, C1 and C2 materials. After all storage times, statistically significant (*p* < 0.0001) differences in the distribution in types of fractures were observed along with an increase in AF, which was related to the increase in the percentage in type A * (24 h) or A (7 days and 30 days). The storage time had a statistically significant effect on the types of fractures of CO material (*p* = 0.0005), which was caused by the appearance of 40% type B fractures after 30 days. There was no statistically significant (*p* > 0.05) influence of storage time on the types of fractures for materials C1, C2 and C4. A statistically significant (*p* < 0.0001) influence of the storage time on the types of fractures for the materials from C6 to C10 was related to the successive increase in the percentage of type A.

**Figure 12:**
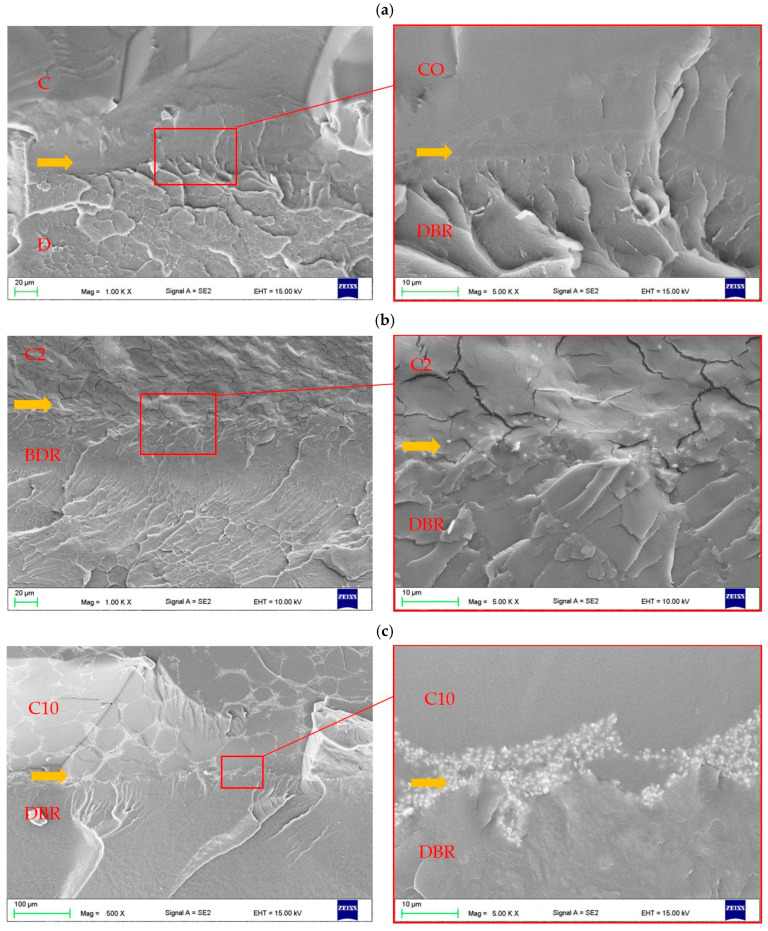
Morphologies of the interface (yellow arrow) between denture base resin and unmodified soft lining material (**a**), C2 (**b**) and C10 (**c**) composites.

**Figure 13:**
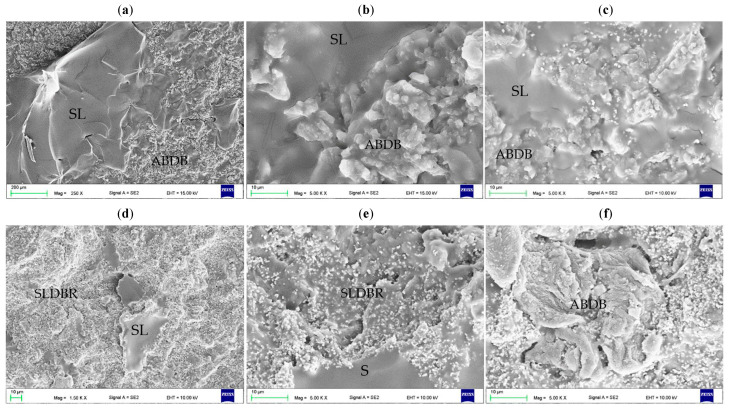
Images (SEM) showing the fractures of the morphologies after tensile bond strength tests for CO (**a**,**b**), C2 (**c**) and C10 (**d**–**f**) materials. The following codes were used to show exemplary areas: SL—soft lining material, ABDBR—alumina-blasted denture base resin (not covered by SL), SLDBR—denture base resin covered by SL.

The SEM morphologies of the DBM interface and soft lining without AF and with AF were presented in Figure 12a–c. For the composites (Figure 12b,c), the layer measuring from approximately 1 to 10 µm (yellow arrow) in direct contact with DBR along its entire length showed the presence of AF particles. Representative SEM images presenting morphologies of fractures after bond strength tests were presented in Figure 13. For CO samples, large areas of morphology obtained after alumina blast without the presence of soft lining material were observed; however, there were also micro and macro areas covered with the soft lining material (Figure 13a,b). As the AF concentration increased, the alumina-blasted surface was covered in an increasing proportion with a layer of composites showing the presence of a large number of AF particles (Figure 13c–e). Uncovered areas, showing the presence of a small number of AF particles on the surface, were still observed (Figure 13f), but for C8 and C10 were rare.

**Figure 14:**
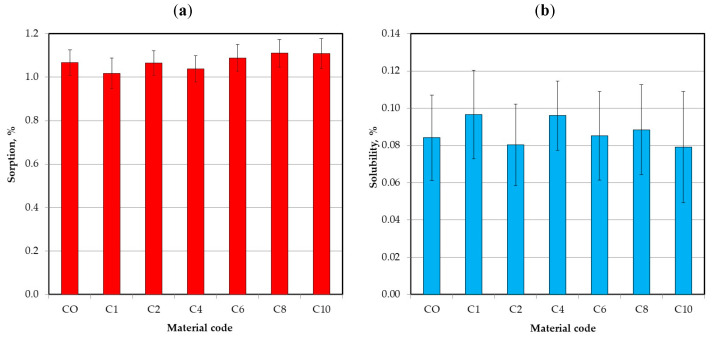
Mean sorption (**a**) and solubility (**b**) with standard deviations for tested materials.

The mean sorption and solubility values are presented in Figure 14. The sorption values ranged from 1.01 to 1.11% and the differences were not statistically significant (*p* = 0.3087). The solubility values were from 0.08 to 0.1% and the influence of AF filler introduction was not statistically significant (*p* = 0.907). The mean sorption and solubility values calculated with EN ISO 10139-2:2016 standard formulas are presented in Figure S1. The sorption values ranged from 12.7 to 14.9 µg/mm^3^ and the solubility were from 1.0 to 1.2 µg/mm^3^.

## 4. Discussion

The purpose of the two-stage procedure of introducing the filler in the matrix (first into the powder and then into the liquid) was to reduce the possibility of aggregation. The advantage of incorporating AF into the powder is to obtain a stable mixture that is easy to store. However, when higher filler concentrations are used, a compact filler layer is formed on the surface of the prepolymerized particles with limited ability to migrate the filler particles into the polymerizing monomer. As a consequence, the dispersion is inhomogeneous and aggregation of the filler is observed in the boundary areas between the prepolymerized particles and the polymer formed from the liquid component, resulting in the formation of structural defects and a reduction in mechanical properties [38]. Limiting the concentration of AF in VSL facilitated rapid mixing of components using ultrasonic homogenization and then modified material handling (lower increase in viscosity of the composition), which was important due the fact of differences in the AF 2.91 g/cm^3^) [31] and VSL (0.95 kg/m^3^), causing a high risk of rapid sedimentation. The dispersion of AF in polymerized samples of materials should be assessed as satisfactory because the formation of filler clusters at the boundary areas created from VSL and developed in the form of VSP was not observed, which proves the correctness of the realized concept. 

During the storage of samples, distilled water was chosen as a medium because the changes in mechanical properties and liquid absorption of dental acrylates in water are greater than after using artificial saliva [39], so it is more restrictive. Moreover, there is no commonly accepted composition of typical artificial saliva used for these types of experiments, and these media are usually recommended for research with metals, not polymer materials. For these reasons, the use of distilled water was the most rational choice in this phase of the experiment. 

The reduction in the number of *C. albicans* colonies for CO in antifungal efficacy tests, which remained in the case of samples stored in distilled water for up to 7 days, at a level ranging from 86 to 97% was probably related to the release into the environment of material components such as peroxides (initiator used in acrylates) or residual monomers that can also exhibit antimicrobial properties [32,40,41]. However, already at the beginning of the experiment the median of CFU/mL for CO was at least 20 times lower than for the composites (4.3 CFU/mL vs. from 0 to 0.3 CFU/mL). In the investigated materials, the concentration of benzoyl peroxide is up to 1% and for other prosthetic materials that contain benzoyl peroxide, the initial antimicrobial efficacy has also been reported [42]. In addition, in the VSL, an addition of stabilizer such as hydrohinone can also be used, which also shows antimicrobial properties [43]. However, these properties were extinguished, as opposed to those observed for experimental composites for which they turned out to be stable in the 30-day period. The AFE values oscillating around 100% after storage suggest that the obtained effect would be maintained for the composites also in the case of extended aging time. Antimicrobial action of the used filler based on ion exchange of cations from the environment (e.g., Na^+^, Ca^2+^) with silver from the insoluble, inorganic carrier, but a controlled release of silver ions is activated under wet conditions [44] so dry samples do not show an antimicrobial effect. The release of silver can eliminate bacteria by varied mechanisms such as a reaction with the peptidoglycan component of the bacterial cell wall causing their puncture, disruption of cellular respiration and metabolic pathways after its penetration into cells, causing the creation of reactive oxygen species due to the disrupted DNA and its replication cycle [45]. The described mechanism of the release of silver ions, determining the antimicrobial properties, indicates that the obtained properties will not only expire with time, but may also depend on the liquid with which the composite is in contact. 

The antifungal efficacy test indicates that the surrounding samples, under dynamic conditions, released the amount of antimicrobial silver ions sufficient to eliminate yeast-like fungi cells in the environment. However, it was equally important to check the ability of a pathogenic microorganism to survive on the tested surfaces in static conditions during an adherence test, where the ions released were not supported by samples/suspension movements because cells may also have deposited during the experiment. These results were less optimistic. The experiment also showed a significantly lower number of living cells adhering to the surface of the composite samples compared to CO, but the reduction in mean values ranged from 67 to 85% (@h h) and from 41 to 69% (30 days). This means that a significant number of cells survived on the surfaces but in their initial state after one month of storage (for CO 14 CFU/mm^2^ and 17 CFU/mm^2^, respectively, and for composites from 2 to 5 CFU/mm^2^, from 5 to 10 CFU/mm^2^, respectively). This situation could be caused by the lower exchange of silver ions with the environment under static conditions, but also by the morphology of the composites characterized by the presence of filler-free areas (Figure 3). The other cause may be the formation of a biofilm composed, among others, of dead cells and components of culture medium. It should be noted that current laboratory experiments are under precisely defined experimental conditions, which is a departure from clinical conditions. The limitation was that the antimicrobial properties of the proposed composites were evaluated after basic storage in distilled water. Due to the discussed mechanism of silver-releasing form filler, the chemical composition of storing liquid may influence antimicrobial properties and its stability. So, in future, additional experiments for chosen filler concentrations should be prepared using an environment characterized by a composition closer to real conditions, e.g., in mucin-containing artificial saliva [46]. Composites should also be investigated in the context of the amount of aggregated biofilm, which formation on soft liners is an essential problem [47]. In the current experiment, we used the basic test based on the suspension of *C. albicans*, which is a promising starting point for further research. Under real conditions, for example, preadsorption on the surface of the salivary proteins, which influence the initial adhesion of microorganisms [48], may also limit the release of silver ions and, in consequence, gradually decrease [49] or even fully inhibit antifungal action. Future experiments with biofilm formation should give a better reference of results to real conditions and look particularly promising if we consider that experimental soft linings with antimicrobial fillers have not been investigated in that context until now. In addition, the available results refer to a biofilm based on *C. albicans* as the most important pathogenic microorganism [50], when synergistic interactions between different microorganisms can also have an influence on the condition of the polymicrobial biofilm [51,52]. 

Due to the fact that the toxicity of silver ions released into the environment has been reported in numerous studies [53] to confirm the beneficial properties of the proposed composites cytotoxicity tests were performed. The cytotoxicity of dental materials containing used filler was not investigated previously. This experiment showed that cell viability for both extraction times exceeded 70%, so none of the materials showed cytotoxic properties [35]. Furthermore, there were no significant differences in cell viability for CO and composite materials. The reduction in viability of cells due to the extension of extracting time was registered for control and experimental materials, which is consistent with other studies on the cytotoxicity of acrylic linings [54], and may be related to the increasing concentration of released components with cytotoxic potential such as plasticizers [55], residual monomers [56] and benzoyl peroxide (initiator) [57].

Most of the previous research on the modification of short-term soft materials linings was related to the introduction of leachable antibiotics or essential oils [20,21]; however the achieved stability was not investigated and the use of antibiotics is criticized due to increasing drug resistance of microorganisms [22]. The use of materials that release metal ions with antimicrobial properties to the environment seems to be of particular interest in this context, because in this case, the mechanisms and the rate of killing the microbial cells prevent the development of mechanisms of resistance to them [58]. It should be emphasized that in the case of prosthetic materials, some works suggest the use of monomers with antimicrobial properties, which, after being incorporated into the polymer network, are not leached [59]. Such a solution seems to be interesting for a durable and stable prevention of colonization of the surface of dentures by microorganisms, but it seems to be less beneficial in the case of supporting the treatment of infected mucosa, because the formation of the salivary film, a deposition of salivary proteins or other substances present in the oral cavity on the surfaces will inhibit the antimicrobial effect in this direction. Moreover, the use of materials with a limited duration of antimicrobial effect is justified because the current direct effect on tissues (e.g., on the condition of mucosa) of continuous exposure to materials with antimicrobial properties is unknown. This risk is not adequately recognized, although there are studies indicating the cytotoxic or genotoxic potential of many materials considered for use in this type of application [60]. The composites should be tested in this respect in the future, although the toxicological data for AF indicate that they should not show cytotoxic properties. [31].

The hardness of soft lining materials is considered to be one of the most clinically important properties due to their influence on the rehabilitation role because softer materials, characterized by lower modulus of elasticity, adapt more easily to the unevenness of the denture bearing area and better distribute the chewing forces transferred to mucosa [61]. The control material and the composites tested showed a hardness similar to that obtained for other soft lining materials [62]. However, the obtained values were lower than those usually registered for acrylic soft lining, including vs. [8] due to the carrying out measurements on samples with a temperature of 37 °C, in contrast to other works, where samples at room temperature were used [8]. Additional measurements for CO samples at room temperature show comparable hardness values than in other works, from 48 Shore A (24 h) to 49.3 Shore A (7 days) [62]. These differences are due to the chemical composition of the starting material used, which determines the glass transition temperature and indicates that the hardness of acrylic soft lining materials should be investigated at their working temperature (~37 °C), because the results obtained in samples at room temperature do not correspond to real functional properties. The dynamics of hardness changes during the experiment for CO and composites was comparable not only to that previously registered for vs. [8,63], but also for those for some silicone long-term soft lining materials [62]. This shows the high stability of the experimental materials and the limited release of components, such as plasticizers, into the environment, which is a disadvantage of many acrylic soft liners [64]. Differences in hardness of CO and composites did not exceed six units on the Shore A scale, indicating that, in practice, these materials are similar. The increase in hardness as a result of the incorporation of fillers into elastomeric polymeric materials was not desirable; however, it was expected because of the reduction in the possible movement of the chains. A similar effect was also observed for other soft lining materials after the introduction of ZnO, TiO_2_ or CeO_2_ [65].

The tests showed a statistically significant increase in tensile strength for the C8 compared to the C10 and a reduction for the C10 compared to the C8 composite. A similar trend was demonstrated by Han et al. [65], where, after introducing ceramic nanofillers, the first increase in tensile strength was recorded for lower contentions and then a decrease was registered. These results remain in agreement with previous reports that ceramic particles can effectively reinforce composites with elastomeric matrix; however, it is important to maintain filler concentration at an appropriate level due to the large specific surface area and high surface energy of the particles [66], which show a tendency to aggregate. This leads to a reduction in the interaction between the particles and the polymer matrix [67] and formation of inhomogeneities in the material, which act as structural defects decreasing the strength properties [68]. These results correspond to the SEM observations of the fractures after the tests. With an increasing AF concentration, the number of faults covered with a large number of particles increased, especially for the C8 and C10 composites. This indicates that the C8 material has reached the filler limit value, above which the negative consequences of its introduction prevail. However, the tensile strength of the C10 material, after the longest storage time, was still statistically significantly higher than that of the CO material. This indicates that elastic matrices present high tolerance to the structural defects created by particles, which stay in accordance with the results by Jabłońska-Stencel et al. [33] for silicone elastomer based composites.

The key property determining the functioning of soft lining materials is the bond strength to the denture base material. A statistically significant effect of the filler introduction on the bond strength was recorded only after 30 days of storage, and the storage time showed a significant effect for C8 and C10. With increasing AF concentration, the higher percentage of A-type fractures increased, and B-type decreased. The SEM observation of fractures showed that disconnection took place for these materials in large areas by the destruction of the layer formed during the polymerization of modified VSL staying in direct contact with the denture base and, consequently, a loss of connection between composites with the denture base material. This zone was subject to preferential destruction which was especially visible in the case of materials from C6 to C10. However, this tendency was registered after 7 days, and was strong after 30 days which may indicate an accelerated degradation of the area of connection, e.g., by migration of water at the boundaries of the AF particle-modified material. This supposition corresponds well to the fact that the decrease in bond strength values for C8 and C10 composites with the simultaneous change in the fracture types was registered only for the longest storage time. An increase in the number of aggregations may additionally intensify this process. Moreover, noted reasons for the decrease in the tensile bond strength are also indicated by the increased or unchanged values in the tensile strength of the composites. 

At the same time, the registered bond strength values for all analyzed composites were sufficient to ensure proper functioning of the lining and correspond to the typical values for this type of material [62].

During immersion in water or saliva from soft denture linings, plasticizers and other soluble components, such us monomers are leached out and water is absorbed due to the properties of polymer net and is expressed in laboratory sorption and solubility tests [69]. Possibly low values for these properties are desirable because of their influence on mechanical properties, stability of dimensions, aesthetic, hygiene and *Candida* growth [70]. The introduction of AF did not cause significant changes in water absorption and solubility, and the original properties of the modified material were retained. These results also correspond well to the results of cytotoxicity tests, where the material extracts showed unchanged properties. The ISO 10139-1:2016 standard for short-term soft lining materials does not require testing for water sorption and solubility; however, ISO 10139-2:2016 for long-term materials establishes a limit for 20 µg/mm^3^ and 3 µg/mm^3^, respectively, so all investigated materials met these requirements. This proves the excellent properties of the tested materials. The values for CO are also consistent with the results obtained by Inoue et al. [71].

Taking into account the fact that aesthetic features are important for dental materials, it should be noted that the introduction of filler was not a cause of brown discoloration, which is typical after nanosilver incorporation [72], but the materials brightened (Figure S2) due to the white color of the filler. A similar effect after the introduction of a silver-releasing ceramic filler was observed by Stencel et al. [73]. In our opinion, the changes were fully acceptable, but, if necessary, they can be removed by the appropriate addition of pigments. Visual assessment did not show any color changes during the experiment, although there is a risk of such changes by silver ions release, their deposition on the surface and oxidation [73].

A limitation is that in the presented initial stage of the experiment, we used static tests for the mechanical properties’ evaluation, which was sufficient, but in future, the influence of the introduced filler on viscoelastic properties should also additionally be investigated. In this regard, complex modulus E* dependent on two components, storage modulus E′ (elastic component of material behavior) with loss modulus E′′ (dissipative component) and the amount of energy dissipated during the cycle expressed by damping factor tan δ (called also loss tangent) are determined. Soft denture liners with relatively high values of tan δ and E′ give better improvements in the increase in masticatory function, but materials having both high cushioning effect and elastic properties have not been developed. Soft linings with a higher tan δ value can absorb more energy during mastication, but on the other hand, worsening elastic recovery during the cyclic mastication due to viscous flow may introduce new “inaccuracies” by deformation of relining which may lead to loss of fit during chewing and less viscous materials can better maintain its softness cycling loading [2].

## 5. Conclusions

The experimental composites, intended for temporary denture soft linings, achieved satisfactory microbiological and mechanical properties during in vitro testing. The most favorable compilation of microbiological and mechanical properties was obtained for composites of C1 to C6 for which nearly complete reduction in *C. albicans* was registered after 30 days of storage of the samples. Adherence of *C. albicans* was significantly reduced compared to CO. The materials showed no cytotoxic potential. The tensile strength and bond strength of these composites were at the desired and stable level and did not differ from the results for the control material or other available results for commercially available materials of this type. Hardness values increased with the increasing concentration of AF, but were within the range typical for this type of soft linings materials and their changes during the experiment were similar to the control material. Sorption and solubility were stable.

## Figures and Tables

**Table 1:** Codes of investigated materials with the concentrations of filler.

Material Code	Final Mass Concentration of AF in Material, %	Mass Concentration of AF in VSP, %	Mass Concentration of AF in VSL, %
CO	0	0	0
C1	1	1	1
C2	2	1	3.3
C4	4	1	7.6
C6	6	1	11.7
C8	8	1	15.6
C10	10	1	19.4

**Table 2:** Antifungal efficacy and the number of viable colonies of *Candida albicans* ATCC 10231 of the test specimen for control material and tested composites stored in distilled water and Kruskal–Wallis test results (α = 0.05) *.

Time		Vt, × 10^2^ CFU/mL							AFE, %						
		CO ^@^	C1 ^#^	C2 ^#^	C4 ^#^	C6 ^#^	C8 ^#^	C10 ^#^	CO	C1	C2	C4	C6	C8	C10
24 h ^@^	Med	4.3	0	0	0	0.3	0	0	95.1	100	100	100	99.7	100	100
	Max	8	0	1	0	0.5	0.5	0.5	96.5	100	100	100	100	100	100
	Min	3	0	0	0	0	0	0	90.7	100	98.8	100	99.4	99.4	99.4
7 d ^@^	Med	4.8	1	0	0	0	0.5	0	94.5	98.8	100	100	100	99.4	100
	Max	11.5	2	0.5	0	0	1	0.5	97.1	100	100	100	100	100	100
	Min	2.5	0	0	0	0	0	0	86.6	97.7	99.4	100	100	98.8	99.4
30 d ^@^	Med	64	0	0.5	0.5	0.3	0	0	25.6	100	99.4	99.4	99.7	100	100
	Max	107	0.5	1	1	2	0	0	52.9	100	100	100	100	100	100
	Min	40.5	0	0	0	0	0	0	−24.4	99.4	98.8	98.8	97.7	100	100

* significantly different results in row or column at the *p* < 0.05 level were marked **^@^**, results that do not differ statistically significantly at the *p* ≥ 0.05 level were marked **^#^**.

**Table 3:** The results of one-way ANOVA and Tukey’s HSD post hoc tests for Shore A hardness *.

Material Code	Storing Time		
	24 h	7 Days	30 Days
	(*p* < 0.0001)	(*p* < 0.0001)	(*p* < 0.0001)
C0 (*p* = 0.05)	A; a	A; a,b	A; b
C1 (*p* = 0.0059)	B; a	B; a,b	B; b
C2 (*p* = 0.1577)	B; -	B; -	B; -
C4 (*p* = 0.1505)	C; -	C; -	C; -
C6 (*p* = 0.0447)	C; a	C; a,b	C; b
C8 (*p* = 0.0221)	D; a	D; a,b	D; b
C10 (*p* = 0.0156)	D; a	E; b	D; b

* The different uppercase letters (A–E) for each column and the lowercase letters (a,b) for each row show significantly different results at the level of *p* < 0.05.

**Table 4:** Results of analysis of variance and Tukey’s HSD post hoc tests for tensile strength *.

Material Code	Storing Time		
	24 h (*p* = 0.1646)	7 Days (*p* = 0.0252)	30 Days (*p* = 0.0002)
C0 (*p* = 0.4161)	-	A	A
C1 (*p* = 0.072)	-	A,B	A
C2 (*p* = 0.1703)	-	A	A
C4 (*p* = 0.1252)	-	A,B	A,B
C6 (*p* = 0.2738)	-	A,B	A,B
C8 (*p* = 0.9173)	-	B	B
C10 (*p* = 0.7359)	-	A,B	A,B

* The different uppercase letters (A,B) for each column show significantly different results at the level of *p* < 0.05.

**Table 5:** Results of analysis of variance and Tukey’s HSD post hoc tests for tensile bond strength *.

Material Code	Storing Time		
	24 h	7 Days	30 Days
	(*p* = 0.169)	(*p* = 0.1799)	(*p* = 0.0038)
C0 (*p* = 0.281)	-	-	A,B; -
C1 (*p* = 0.7639)	-	-	A,B; -
C2 (*p* = 0.4551)	-	-	B; -
C4 (*p* = 0.0777)	-	-	A,B; -
C6 (*p* = 0.0581)	-	-	A,B; -
C8 (*p* = 0.0381)	a	a,b	A; b
C10 (*p* < 0.0001)	a	b	A; c

* The different uppercase letters (A,B) for columns and lowercase letters (a–c) for rows show significantly different results at the level of *p* < 0.05.

## Data Availability

The datasets used and/or analyzed during the current study are available from the corresponding author on reasonable request.

## Supplementary Materials

The following supporting information can be downloaded at: https://www.mdpi.com/article/10.3390/ma17040902/s1, Figure S1: Photograph of samples of the control material and composites in their initial state and after conditioning, visible progressive lightening of the materials after the introduction of the filler. Figure S2: Mean sorption (a) and solubility (b) calculated uising EN ISO 10139-2:2016 formulas (w_sp_ is sorption, w_sl_ is solubility, m_l_ is the initial mass of dried sample, µg; m_2_ is the mass after storing, µg, and m_3_ is the mass after the second drying, µg and V is the volume of the sample, mm^3^).
