# Supplementary material for: A Temporary Acrylic Soft Denture Lining Material Enriched with Silver-Releasing Filler-Cytotoxicity, Mechanical and Antifungal Properties
Source: Materials (Basel). 2024 Feb 15;17(4):902. doi: 10.3390/ma17040902 (PMC10890124; doi:10.3390/ma17040902)
Supplement: Supplementary file 1 [file materials-17-00902-s001.zip › materials-2844335-supplementary.pdf]

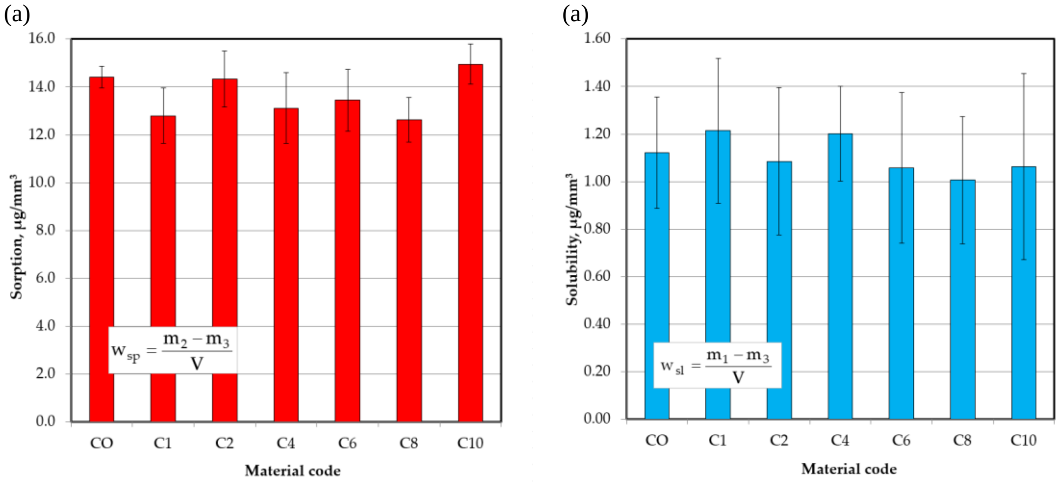

Figure S1. Mean sorption (a) and solubility (b) calculated uising EN ISO 10139-2:2016 formulas (w_{sp} is sorption, w_{sl} is solubility, m_l is the initial mass of dried sample, μg; m_2 is the mass after storing, μg, and m_3 is the mass after the second drying, μg and V is the volume of the sample, mm^3).

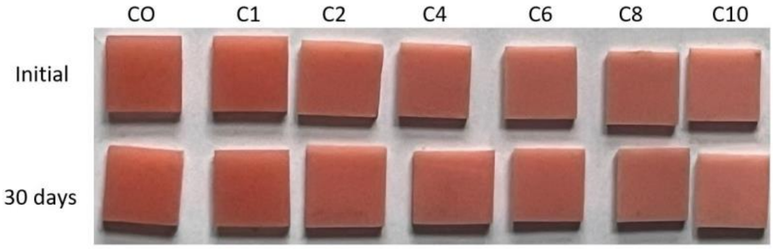

Figure S2. Photograph of samples of the control material and composites in their initial state and after conditioning, visible progressive lightening of the materials after the introduction of the filler.
